# Sustained Release of Hydrophilic l-ascorbic acid 2-phosphate Magnesium from Electrospun Polycaprolactone Scaffold—A Study across Blend, Coaxial, and Emulsion Electrospinning Techniques

**DOI:** 10.3390/ma7117398

**Published:** 2014-11-17

**Authors:** Xinxin Zhao, Yuan Siang Lui, Pei Wen Jessica Toh, Say Chye Joachim Loo

**Affiliations:** 1 School of Materials Science and Engineering, Nanyang Technological University, 50 Nanyang Avenue, 639798 Singapore; E-Mails: xinxin.zhao@gmail.com (X.Z.); YLUI001@e.ntu.edu.sg (Y.S.L.); jessica-toh@imre.a-star.edu.sg (P.W.J.T.); 2 Singapore Centre on Environmental Life Sciences Engineering (SCELSE), Nanyang Technological University 60 Nanyang Drive, 637551 Singapore

**Keywords:** electrospinning, ascorbate-2-phosphate, release

## Abstract

The purpose of this study was to achieve a sustained release of hydrophilic l-ascorbic acid 2-phosphate magnesium (ASP) from electrospun polycaprolactone (PCL) scaffolds, so as to promote the osteogenic differentiation of stem cells for bone tissue engineering (TE). ASP was loaded and electrospun together with PCL via three electrospinning techniques, *i.e.*, coaxial, emulsion, and blend electrospinning. For blend electrospinning, binary solvent systems of dichloromethane–methanol (DCM–MeOH) and dichloromethane–dimethylformamide (DCM–DMF) were used to achieve the desired ASP release through the effect of solvent polarity and volatility. The scaffold prepared via a blend electrospinning technique with a binary solvent system of DCM–MeOH at a 7:3 ratio demonstrated a desirable, sustained ASP release profile for as long as two weeks, with minimal burst release. However, an undesirable burst release (~100%) was observed within the first 24 h for scaffolds prepared by coaxial electrospinning. Scaffolds prepared by emulsion electrospinning displayed poorer mechanical properties. Sustained releasing blend electrospun scaffold could be a good potential candidate as an ASP-eluting scaffold for bone tissue engineering.

## 1. Introduction

Autologous bone grafts are often the gold standard for reconstructing bone defects after injury or tumor resection. However, their limited availability and the associated donor site morbidity restrict their wide usage in clinical practice [1,2]. Tissue engineering (TE) strategies such as developments of scaffolds have thus emerged as alternative treatments to improve on these aforementioned issues. A critical factor for bone TE materials is the possession of good mechanical properties, while having the ability to provide a temporal and spatial presentation of growth factors and osteogenic supplements. For example, in order to effectively trigger osteogenesis and bone remodeling, growth factors or supplements have to be loaded into these scaffolds and released in a timely and controlled manner.

For TE applications, the supply of therapeutic compounds was therefore considered to be important because this could provide the right stimulation to cells, allowing them to differentiate and proliferate in an optimal manner. For instance, Jabbari *et al*. demonstrated sustained releasing rhBMP2 could have higher osteopontin expression level in bone mesenchymal stem cells compared to the direction of rhBMP2 (a 4-fold increase within 21 days of culture) [3]. As for dexamethasone (DEX), a synthetic glucocorticoid, it has been shown that contrasting observations on osteoblast cultures can occur that are highly dependent on the dosage supplied [4]. Therefore, in order to promote proper bone healing, a good delivery scaffold should release supplements adequately and sustainably at a desirable rate and profile, whereby 40–400 ng/mL is required over three weeks.

l-ascorbic acid 2-phosphate magnesium (ASP), also known as vitamin C, exhibits many attractive properties and is therefore widely used for biological, dermatological and pharmaceutical purposes [4]. ASP essentially aids in improving cell viability and stimulates the production of collagen, alkaline phosphatase and DNA biosynthesis [5,6]. However, ASP, which cannot be synthesized by human and non-human primates, must be supplied exogenously and transported intracellularly to the cell membranes [7,8]. Approximately 16–160 µg/mL of dosage for two weeks is required to adequately induce osteogenic differentiation. While the controlled release of DEX from different drug carriers has been reported [9,10,11,12], the controlled release of ASP is far less explored. From the limited reports so far, the release of ASP has generally been shown to be short term (less than three days), regardless of the carrier type, *i.e.*, nano-capsules [13], micro-capsules [6], or polymer scaffolds [14]. This is due to its low molecular weight and high water solubility, making it a difficult drug with which to achieve both controlled and sustained release. Even though ASP has been shown to be released for up to 60 days, as reported by Zhang *et al*. [1], the release amount (~2.5 µM per day) was found to be much less than the required dosage (~20 µM) to induce osteogenic differentiation. Moreover, the chemical bonding reaction used, as reported, had to be in a nitrogen environment for five days in order that the chemical bonds were properly formed.

Of the various techniques used for the synthesis of scaffold materials, electrospun materials have been shown to be highly promising, especially in obtaining three dimensional (3D) artificial bone grafts for bone TE. Electrospun scaffolds possess extracellular matrix (ECM)-mimicking features that can facilitate cell adhesion, differentiation and proliferation, as well as provide structural support to the defect. Notably, nanofibrous scaffolds fabricated via electrospinning techniques have been extensively reviewed for their potential as carriers for therapeutic compounds. Based on solvent preparation and other parameters, electrospinning techniques can be generally classified as blend, emulsion and core-sheath [15,16,17]. In spite of the convenient incorporation of therapeutic compounds into nanofibers in a single-step process (blend electrospinning), burst release was inevitable especially for hydrophilic drugs such as ASP due to their incompatibility with the polymeric matrix. To overcome this, different formulations and set ups have been designed to form a biphasic suspension (emulsion electrospinning) or a core-sheath biphasic fibrous structure (coaxial electrospinning) in order to avoid the initial burst release and provide better sustained release [18].

In this study, we investigated the feasibility of achieving sustained release of ASP through different electrospinning techniques, namely blend, emulsion and coaxial electrospinning. The release profiles and the mechanical properties of the respective scaffolds were also studied. Here, it is shown for the first time, how the sustained release of ASP, at an effective daily amount [19] required for osteogenic differentiation of stem cells, could be achieved from electrospun scaffolds.

## 2. Results and Discussion

For bone TE, an ideal scaffold should mimic the natural extracellular matrix by providing adequate mechanical support and the necessary biochemical factors. The physical properties of the scaffold might influence cell attachment, proliferation and differentiation, while the biochemical cues might further enhance or regulate specific cellular responses in a time-dependent manner. To induce proper osteogenic differentiation, sufficient ASP (50 µM every 2–3 days) has to be supplied continuously for 2–3 weeks. However, due to the hydrophilic nature and low molecular weight, long term controlled release of ASP at the desired daily release amount (~20 µM/per day) from a hydrophobic matrix remains a challenge to date. For example, despite the fact that ASP has been reported to release for up to 40 days *in vitro* from PLGA porous scaffolds [20], more than 50%–70% of ASP was released through an initial burst. Moreover, the continuous daily release amount of 0.017–0.269 µg was far less than the required dosage. In order to increase the required daily release amount, the ASP loading amount was increased by nine times to ~1 wt% in the follow up *in vivo* test [21]. As such, more than 95% of burst release was observed within the first seven days. Zhang *et al*. [1] reported scaffold degradation controlled sustained release of ascorbic acid from a three dimensional polyurethane matrix. Here, because ASP was chemically bonded with the polymeric matrix, the drug release rate and amount were restricted by the degradation rate of the scaffold and hence did not provide sufficient stimulation to the cells [1].

### 2.1. ASP–PCL Scaffolds Prepared by Coaxial and Emulsion Electrospinnings

**Figure 1:**
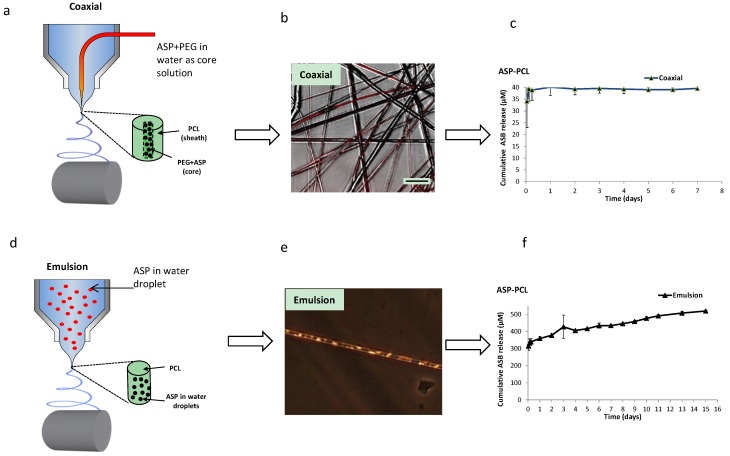
(**a**) A schematic overview of ascorbate-2-phosphate (ASP)–electrospun polycaprolactone (PCL) microfibers prepared by coaxial; (**b**) corresponding confocal image: inner core was propidium iodine (PI) stained; (**c**) cumulative ASP release profile; (**d**) a schematic overview of ASP–PCL microfibers prepared by emulsion electrospinning method; (**e**) phase contrast image to show homogeneous distributed ASP aqueous droplets in PCL fibers; (**f**) cumulative ASP release profile of scaffolds prepared by emulsion electrospinning. Scale bars represent 10 µm.

To resolve the burst release issue especially for hydrophilic drugs, coaxial and emulsion electrospinning setups had been researched previously [15], and were used as a control in this study (Table 1). For coaxial electrospinning, electrospun polycaprolactone (PCL) solution and ASP in a PEG (polyethylene glycol) aqueous core solution were injected through the nozzles and electrospun to give core-sheath fibers (Figure 1a). The core-sheath structure of the fibers was determined by using propidium iodide (PI) to fluorescently stain the core region red (Figure 1b). Even though the ASP was localized into the core region, nearly 100% of ASP was released out within 24 h (Figure 1c). This undesirable burst release could be attributed to the “edge effect” of the electrospun scaffolds [22]. Because of the cutting edges of the coaxial electrospun samples, ASP located in the continuous core was directly exposed to PBS (phosphate buffered saline) and hence resulted in the fast release.

Figure 1d illustrates the formation of ASP (aqueous)–PCL (oil) emulsion and the corresponding emulsion electrospinning. PCL was dissolved in dichloromethane (DCM) to form the continuous phase, while ASP was dissolved in aqueous solution to form the dispersed phase. Figure 1e displays that micro-sized ASP droplets (which appeared as bright spots within the fibers) were randomly distributed in the PCL fibers, with fiber diameter ranging from 1 to 5 µm. The ASP reservoirs were protected by the hydrophobic PCL barrier from the outer release environment (PBS). Therefore, the release profile of ASP from emulsion scaffolds lasted for 15 days, showing nearly zero-order release kinetics (Figure 1f), after 359.1 µM (~50%) of burst release on the first day. However, a relatively high burst release (~40%) was still observed, and this could be due to the ASP absorbed on fiber surfaces because of the bursting of water droplets during the drying process.

**Table 1:** Scaffold information.

Scaffolds (Electrospun methods)		Loading efficiency (%)	Initial release within day 1 (%)	Total release within 14 days (%)	Young’s Modular (MP)
Coaxial		92.6 ± 3.4	86.1 ± 15.3	99.8	-
Emulsion		46.1 ± 7.2	42.5 ± 2.7	70.2	8.7
Blend	DCM–DMF = 7:3	78.5 ± 9.7	7.8 ± 0.3	15.2	9.7
	DCM–MeOH = 8:2	73.7 ± 1.5	26.9 ± 0.9	50.2	6.7
	DCM–MeOH = 7:3	80.3 ± 4.5	22.0 ± 1.9	59.1	6.7
	DCM–MeOH = 6:4	81.0 ± 11.2	23.2 ± 4.6	55.4	6.4

Dichloromethane (DCM), Dimethylformamide (DMF).

### 2.2. ASP–PCL Scaffolds Prepared by Blend Electrospinning by Using Binary Solvent Systems

**Figure 2:**
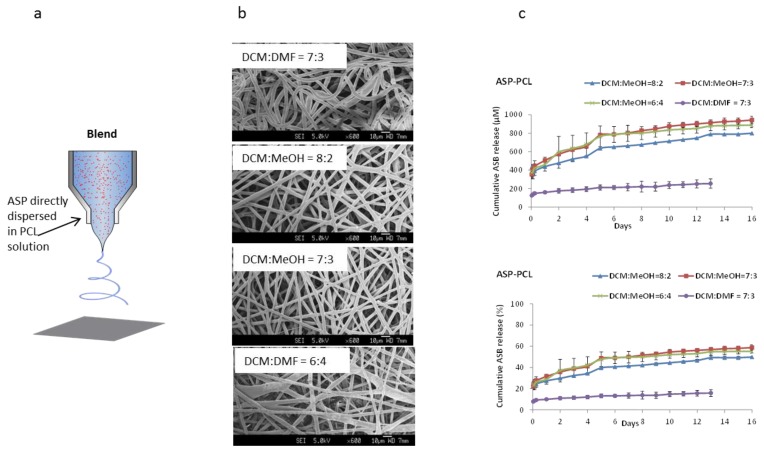
(**a**) Scheme of blend electrospinning methods; (**b**) SEM (scanning electron microscope) images of ASP–PCL scaffolds prepared by blend electrospining using binary solvent systems; and (**c**) Cumulative release of ASP from blend electrospun scaffolds. Scale bars represent 10 µm.

For blend electrospinning, DCM was used as non-polar solvent to dissolve PCL, while MeOH and DMF were used as polar solvent to contain ASP and to adjust the electrospinnability of PCL solution. Figure 2a shows a schematic of how ASP was blended with PCL in different binary solvent systems. From the SEM images (Figure 2b), it can be seen that continuous and randomly distributed mesh-scaffolds were successfully electrospun for all formulations. However, fiber morphologies of the scaffolds obtained under different formulations varied significantly. For example, compared with the scaffold produced from the DCM–DMF systems which appeared “curly”, a more stretched fiber morphology was observed for DCM–MeOH scaffolds. This may due to the slower drying speed because of the low volatility of DMF (b.p. 153 °C) as compared with that of MeOH (b.p. 64.7 °C) (Figure 2b). Furthermore, the dielectric constant of the solvents has a significant impact on the structure of the resulting fibers [23]. The higher dielectric constant of DMF (38.3) compared with that of methanol (33) facilitated the formation of curled fibers with smaller diameters [24]. Further increasing the percentage of MeOH resulted in a thinner fiber diameter. However, the distribution of fiber diameters became inhomogeneous upon further increasing the percentage of MeOH to 40% (DCM:MeOH = 6:4), which may due to the high viscosity of the electrospun solution induced by the poor solubility of PCL with the increased MeOH ratio [25]. The ASP release profiles of the blend scaffolds were studied over two weeks and compared (Figure 2c). Solvent type and ratio dependent ASP releases were observed despite the trivial differences in the scaffold morphologies. The DCM–DMF system had relatively low release on the first day (8%), and an insignificant amount released in the following 14 days. In contrast, ASP was sustained released for 14 days in the DCM–MeOH systems even after 20% of release on the first day. The difference in ASP release profiles of the DCM–MeOH and DCM–DMF binary solvent systems might be attributed to the ASP distribution inside the fibers. PCL, which is hydrophobic, would be preferentially dissolved in DCM, while the ASP was preferentially dissolved in DMF or MeOH. During the fiber formation process, DCM (boiling point = 39.6 °C) evaporated first to form a “PCL barrier”. Subsequently, ASP, which preferentially interacts with MeOH or DMF, would be entrapped inside by the formed PCL barrier. The PCL barrier around ASP might have contributed to the lower burst release for blend electrospun scaffolds. With increasing MeOH ratio in DCM–MeOH systems, the conductivity of the binary solvents increased, resulting in fibers with smaller diameter. The decreased fiber diameter facilitated faster ASP release due to the higher specific surface area. Nevertheless, desirable ASP release was achieved through both emulsion and blend electrospun scaffolds.

### 2.3. Mechanical Properties of ASP–PCL Scaffolds

**Figure 3:**
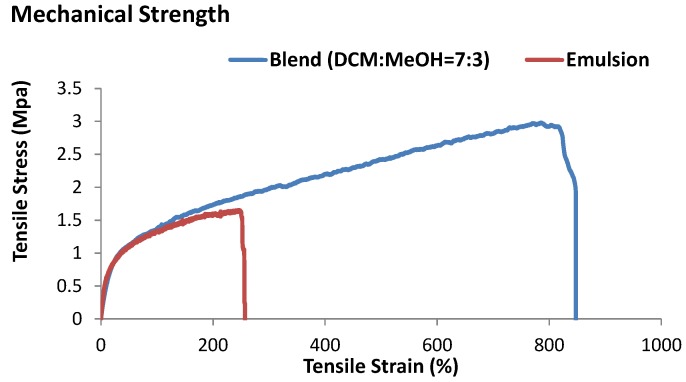
Comparison of mechanical strength of the scaffolds produced by different electrospinning methods.

Tensile measurements were subsequently conducted for emulsion and blend electrospun scaffolds. From Figure 3, the tensile strength (TS) and strain at break (ε) of the scaffolds were 1.5 ± 1.0 MPa and 250% for scaffold prepared by the emulsion method, and 3.0 ± 0.5 MPa and 847% for scaffolds prepared by the blend method. The decrease in both tensile stress and the strain to break value for emulsion electrospun scaffold compared with that of blend electrospun scaffold, suggested that the scaffolds prepared by the emulsion method are less suitable for bone TE as they do not ensure sufficient mechanical support. The low mechanical strength of emulsion electrospun scaffold could be because of micropores forming in the fibers, serving as mechanical defects [26,27]. The mechanical strength of the scaffolds could be improved by decreasing the emulsion size and improving the dispersity of the emulsion droplets inside the fibers.

## 3. Experimental Section 

All chemicals and reagents utilized in this study were purchased from Sigma-Aldrich Inc. (St. Louis, MO, USA) and used as received, unless otherwise stated. 

### 3.1. Scaffold Preparation

#### 3.1.1. Preparation of ASP–PCL Solutions for Coaxial, Emulsion and Blend Electrospinning

For coaxial electrospinning, 12 *w*/*v*% of PCL solution (sheath solution) in DCM–DMF co-solvents in the ratio of DCM:DMF = 7:3 was prepared by dissolving PCL (1.2 g) in 7 mL of DCM and 3 mL of DMF. The aqueous core solution was prepared by dissolving 30 mg PEG together with 2 mg ASP in 1 mL deionized (DI) water.

For emulsion electrospinning, 14 *w*/*v*% of PCL solution was prepared by dissolving PCL (2.1 g) and Span 80 (23 mg) in 15 mL DCM. Separately, the ASP solution was prepared by dissolving ASP (126 mg) and PEG (105 mg) in 1.5 mL of DI water. ASP aqueous solution (0.75 mL) was subsequently added into PCL solution in a dropwise manner, followed by vigorous stirring for 10 min and sonication for 15 s. The solution was stirred overnight and re-homogenized by stirring and sonication before electrospinning.

For blend electrospinning, DCM–DMF and dichloromethane-methanol (DCM–MeOH) binary solvent systems were used. PCL (14 *w*/*v*%) was dissolved in DCM–DMF and DCM–MeOH respectively, with a mixing ratio of 7:3 to investigate the effect of the type of co-solvent. For DCM–MeOH systems, other than the ratio of 7:3 (DCM:MeOH), different ratios of 8:2 and 6:4 were also investigated to see the effect of the solvent ratio. The electrospinning solutions were prepared by directly adding ASP (42 mg) into 10 mL of 14 *w*/*v*% PCL solutions. Solutions were stirred overnight to ensure complete homogeneous dissolution and were subsequently electrospun.

#### 3.1.2. Electrospinning of ASP–PCL Scaffolds

Electrospinning was performed with the Nanon-01A (MECC, Fukuoka, Japan). For coaxial electrospinning, the PCL solution and ASP solution were separately delivered by two syringe pumps to the outer and inner tube with controlled flow rates of 6 and 0.6 mL/h, respectively. The voltage applied was 18.5 kV. The scaffold was collected by a drum collector (500 rpm) at a distance between needle tip and collector of 14 cm.

For emulsion and blend electrospinning, polymer solutions were electrospun by using a blunt-tip 18 gauge needle at a constant feed rate of 10 mL/h. For emulsion electrospinning, the applied voltage was initially set at 18 kV but the voltage was changed to 16.5 kV after stable flow was observed. The scaffolds were collected on a drum collector (500 rpm) at a distance between the needle tips to the collector of 14 cm. For blend electrospinning, the voltage was 16.5 kV and the scaffold was collected by a flat collector at distance of 14 cm.

### 3.2. Scaffold Characterization

#### 3.2.1. Morphology Assessment 

The core-sheath structure of fibers prepared by coaxial electrospinning was confirmed by using a confocal microscope (Leica TCS SP5, Leica Microsystem, Wetzlar, Germany). In order to observe the distribution of ASP in the core-sheath fibers, hydrophilic fluorescent dye (propidium iodide: PI) loaded scaffolds were prepared. PI was added in the ASP aqueous core solution at a concentration of 1 µg/mL and electrospun together with the PCL sheath solution. The fibers as obtained were vacuum dried overnight in the dark before imaging. For emulsion electrospinning, fibers were collected on glass microscope slides and imaged using an inverted phase contrast light microscope (Nikon Eclipse-Ti^TM^, Nikon instruments Inc., Melville, NY, USA) at 200× magnification. The morphologies of the blend electrospun scaffolds were observed under Field Emission Scanning Electron Microscopy (FESEM) (JSM-6340F, JEOL Co., Tokyo, Japan) at an operation voltage of 5 kV. Before analysis, the samples were coated with platinum at 20 mA for 60 s. Mean fiber diameters were determined by measuring 50 independent fibers using Image J software (version 1.48) (*n* = 50) (see Figure S1).

#### 3.2.2. ASP Encapsulation Efficiency

Encapsulation efficiency was defined as the ratio of actual to theoretical drug loading within the scaffolds. Theoretically, 3 wt% of ASP was loaded into the ASP–PCL scaffolds. In order to quantify the ASP amount loaded in PCL scaffolds obtained through the aforementioned electrospinning setups, ASP–PCL scaffolds were cut and weighed before dissolving in DCM. ASP was then extracted by DI water, into which the hydrophilic drug preferentially partitioned. This ASP aqueous solution was then freeze dried and re-dissolved into 10 mL DI water. The concentration of ASP was determined using a UV-Vis spectrophotometer (Shimadzu UV-2501, Kyoto, Japan) at 260 nm. All measurements were done in triplicate (*n* = 3).

#### 3.2.3. ASP Release Study

Scaffolds (ASP–PCL) were cut and weighed, in triplicate, before being transferred into vials containing 5 mL PBS (PAA Laboratories GmbH, Pasching, Austria). These vials were maintained at 37 °C in a shaking incubator. At pre-determined time intervals, 1 mL of medium from each vial was removed and replenished with fresh PBS solution. The solutions were analyzed by using a Shimadzu UV-2501 UV-Vis spectrophotometer at λ_ASP_ = 260 nm. 

#### 3.2.4. Mechanical Properties

The mechanical properties of the scaffolds were evaluated at room temperature using the Instron Tensile Machine 5567 (Instron, Canton, MA, USA). American Society for Testing and Materials (ASTM) dog bone shaped specimens of dimension 9.53 mm × 3.18 mm were prepared and tested using a load cell of 500 N at a tensile loading rate of 10 mm·min^−1^. The mechanical properties of the scaffolds were calculated (BlueHill version 2.21) for their respective tensile strength (TS), strain at break (ε) and Young’s modulus (*E*) in triplicate (*n =* 3).

### 3.3. Statistical Analysis

All data were expressed as mean ± standard deviation. Statistical differences were determined with student’s *t*-test and differences were statistically significant at *p* ≤ 0.05.

## 4. Conclusions

ASP was loaded and electrospun together with PCL by coaxial, emulsion and blend electrospinning methods. A burst release (~100%) was observed from scaffolds prepared by coaxial electrospinning, which could be attributed to the “edge effect”. The burst release of ASP was avoided, and a sustained release of ASP with an effective daily amount (~20 µM/day) was achieved for two weeks, through the scaffolds prepared by emulsion and blend electrospinning. The controlled and sustained release of ASP, together with its higher mechanical properties, make ASP–PCL scaffolds prepared by the blend electrospinning method using DCM–MeOH as the binary solvent system more promising.

## Supplementary Materials

Supplementary materials can be accessed at: http://www.mdpi.com/1996-1944/7/11/7398/s1.
