# Supplementary material for: Sustained Release of Hydrophilic l-ascorbic acid 2-phosphate Magnesium from Electrospun Polycaprolactone Scaffold—A Study across Blend, Coaxial, and Emulsion Electrospinning Techniques
Source: Materials (Basel). 2014 Nov 17;7(11):7398–408. doi: 10.3390/ma7117398 (PMC5512642; doi:10.3390/ma7117398)

Supplementary Materials

Figure S1. Comparison of fiber diameters of the scaffolds produced through different electrospinning techniques (*n* = 50).

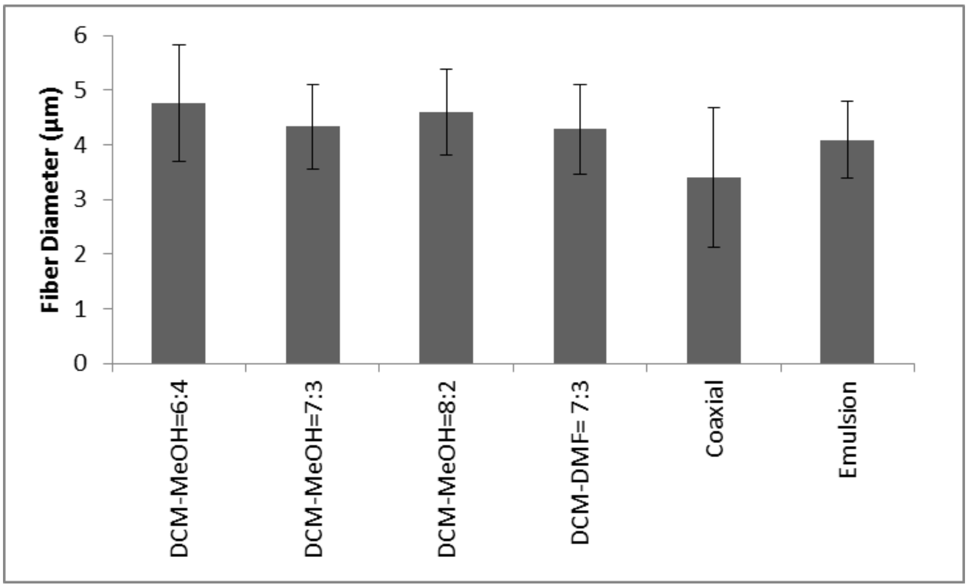

Supplement: Supplementary file 1 [file materials-07-07398-s001.pdf]
